# Complex conjugate removal in optical coherence tomography using phase aware generative adversarial network

**DOI:** 10.1117/1.JBO.30.2.026001

**Published:** 2025-02-17

**Authors:** Valentina Bellemo, Richard Haindl, Manojit Pramanik, Linbo Liu, Leopold Schmetterer, Xinyu Liu

**Affiliations:** a Nanyang Technological University, School of Chemistry, Chemical Engineering and Biotechnology, Singapore, Singapore; b Singapore Eye Research Institute, Singapore National Eye Centre, Singapore, Singapore; c SERI-NTU Advanced Ocular Engineering, Singapore, Singapore; d Medical University of Vienna, Center for Medical Physics and Biomedical Engineering, Vienna, Austria; e Iowa State University, Department of Electrical and Computer Engineering, Ames, Iowa, United States; f Guangzhou National Laboratory, Guangzhou, Guangdong, China; g Duke-NUS Medical School, Ophthalmology and Visual Sciences Academic Clinical Program, Singapore, Singapore; h Institute of Clinical and Experimental Ophthalmology, Basel, Switzerland; i Medical University of Vienna, Department of Clinical Pharmacology, Vienna, Austria; j Peking University, Institute of Medical Technology, Beijing, China

**Keywords:** complex conjugate removal, optical coherence tomography, generative adversarial networks

## Abstract

**Significance:**

Current methods for complex conjugate removal (CCR) in frequency-domain optical coherence tomography (FD-OCT) often require additional hardware components, which increase system complexity and cost. A software-based solution would provide a more efficient and cost-effective alternative.

**Aim:**

We aim to develop a deep learning approach to effectively remove complex conjugate artifacts (CCAs) from OCT scans without the need for extra hardware components.

**Approach:**

We introduce a deep learning method that employs generative adversarial networks to eliminate CCAs from OCT scans. Our model leverages both conventional intensity images and phase images from the OCT scans to enhance the artifact removal process.

**Results:**

Our CCR-generative adversarial network models successfully converted conventional OCT scans with CCAs into artifact-free scans across various samples, including phantoms, human skin, and mouse eyes imaged *in vivo* with a phase-stable swept source-OCT prototype. The inclusion of phase images significantly improved the performance of the deep learning models in removing CCAs.

**Conclusions:**

Our method provides a low-cost, data-driven, and software-based solution to enhance FD-OCT imaging capabilities by the removal of CCAs.

## 1 Introduction

In most Fourier-domain optical coherence tomography (OCT) devices, only half of the available depth range is used due to complex conjugate ambiguity, an artifact arising from the symmetrical mirror about the zero-delay position of the B-scan image.^1^ The typical approach to avoid complex conjugate artifacts (CCAs) is to reduce half of the imaging range so that the region of interest within the sample exclusively resides on one side of the zero delay. Overcoming this ambiguity and generating full-depth tomograms would double the imaging depth.^2^

Presently, various methods have been proposed to address this challenge, including phase-shifting interferometry,^3^ dispersion encoding for spectral domain OCT,^4^ heterodyne detection for swept-source OCT (SS-OCT),^5^ polarization optics,^6^ and passive quadrature demultiplexing.^7^ However, these techniques need the inclusion of supplementary active or passive components, thereby increasing system complexity and computational costs. Deep learning (DL) has revolutionized the methodologies of imaging processing and has enabled significant progress in the field of medical imaging.^8^ The generative adversarial network (GAN) is a powerful DL architecture to edit and translate images in two domains by recursively training a generator to produce synthetic images against a discriminator for distinguishing between synthetic and real images.^9^ In OCT imaging, GANs have been reported for image denoising,^10^^,^^11^ super-resolution,^12^ and generating realistic retinal scans for medical education and data augmentation.^13^

In this article, we propose to train a complex conjugate removal GAN (CCR-GAN) network to translate B-scans with CCA to their paired images without CCA. We collected the paired image data using a customized OCT system on various samples, including polymer phantoms, human skin, and mouse eyes. We demonstrated the effectiveness of our CCR-GAN in eliminating CCA for *in vivo* OCT images and evaluated the outputs quantitatively using similarity metrics, providing a more refined understanding from previous work.^14^ Furthermore, we found that the phase information of OCT scans improves the performance of the GAN models in this task.

## 2 Methods

### 2.1 Optical Setup and Data Acquisition

**Fig. 1:**
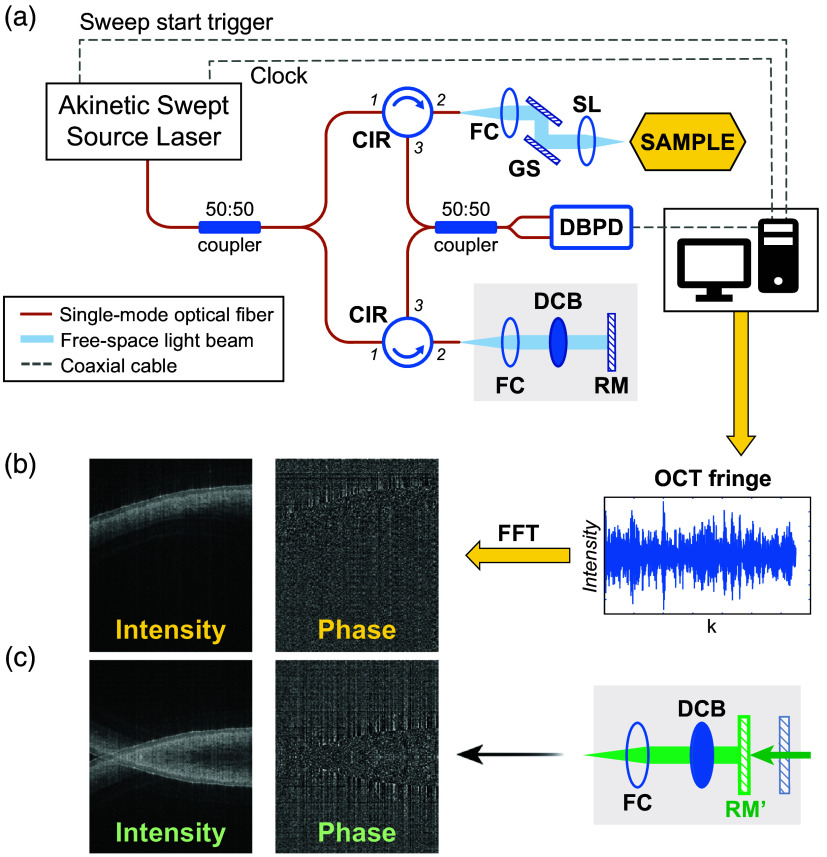
(a) System schematic of the SS-OCT setup. (b) Ground truth OCT data acquisition. (c) Reference mirror shift to place the sample at the zero-delay position and acquire input OCT data with CCAs. CIR, optical circulator, FC, Fiber collimator, GS, Galvo scanning mirror, SL, Scan lens, DBPD, Duel balanced photodetector, DCB, Dispersion compensation block, RM, Reference mirror, and FFT, Fast Fourier transform.

The SS-OCT [Fig. 1(a)] used for data acquisition in this study was a fiber-based system utilizing a semiconductor phase-stable light source (Insight Photonic Solutions Inc., Lafayette, Colorado, United States) with a sweep rate of 100 kHz, a central wavelength of 1300 nm, and a bandwidth of 90 nm. The theoretical axial resolution was around 8  μm in tissue, and the measured lateral resolution was around 20  μm.

As a proof-of-concept setup to acquire paired images with and without CCAs, we used a 1-GHz high-speed digitizer to ensure the imaging range of this system is more than 5 mm in terms of 3-dB sensitivity rolling-off. The measured sensitivity was 120 dB with a radiant power of 20 mW on the surface of the sample. The beam was scanned using two galvanometer mirrors (GVS002, Thorlabs GmbH, Dachau, Germany) and focused onto the sample through a 36-mm focal length scan lens (LSM03, Thorlabs GmbH, Dachau, Germany).

To obtain the dataset to train and evaluate the DL network, we prepared three kinds of samples, polymer phantoms made of multilayer acrylonitrile butadiene styrene sheets (McMaster-Carr, 5751T51), hands *in vivo* from two volunteers, and eyes *in vivo* from three anesthetized mice (outbred albino IcrTac:ICR, female). Each volumetric scan comprised 100, 200, or 400 B-scans with OCT scanning over 6 mm by 1, 2, or 4 mm areas, respectively. The intensity B-scans were obtained from the magnitude of the fast Fourier transform (FFT) of the acquired OCT interference fringes and were converted to logscale and normalized to 0 to 255 to produce the grey-scale images. In addition, as the phase images resulting from the FFT contained symmetrical artifacts that were similar to those in the intensity images, we also investigated inputting the phase images to the CCR-GAN model as another input channel. To input the phase image, we converted the phase maps (0−2π) to gray-scale (0–255).

We initially used half of the depth range to acquire the paired images with and without CCA. Then, we placed the sample image in the optimal depth position, such as in a conventional OCT [Fig. 1(b)]. After a volumetric scan, without moving the sample, we moved the reference mirror to place the sample image exactly on the zero-delay position [Fig. 1(c)] where the CCAs were obviously visible and then performed a second volumetric scan. In this way, a dataset with paired images with and without CCA was obtained. Matching the depth location in the paired images is not necessary in these datasets for GAN models.

For the polymer phantoms, we acquired 19 paired volumetric scans from three distinct phantoms from randomly varying locations. For human skins, we acquired 19 paired volumetric scans on different skin locations of the hands from two volunteers, including the fingertip and palm (Nanyang Technological University Institutional Review Board IRB-2016-10-015). For the animal eyes, we anesthetized three mice^15^ indexed by serial numbers (S/N) 1 to 3 and scanned their eyes at the surface of the cornea with slightly varying illumination angles. As the mouse eye is small, we can see the whole eye structure including the cornea, iris, and retina in a single B-scan. All procedures in this study adhered to the Association for Research in Vision and Ophthalmology (ARVO) statement for the use of animals in ophthalmic and vision research and were approved by the Institutional Animal Care and Use Committee, Nanyang Technological University, Singapore (Protocol No.: A19101).

### 2.2 Deep Learning Model Architecture, Training, and Testing Strategies

**Fig. 2:**
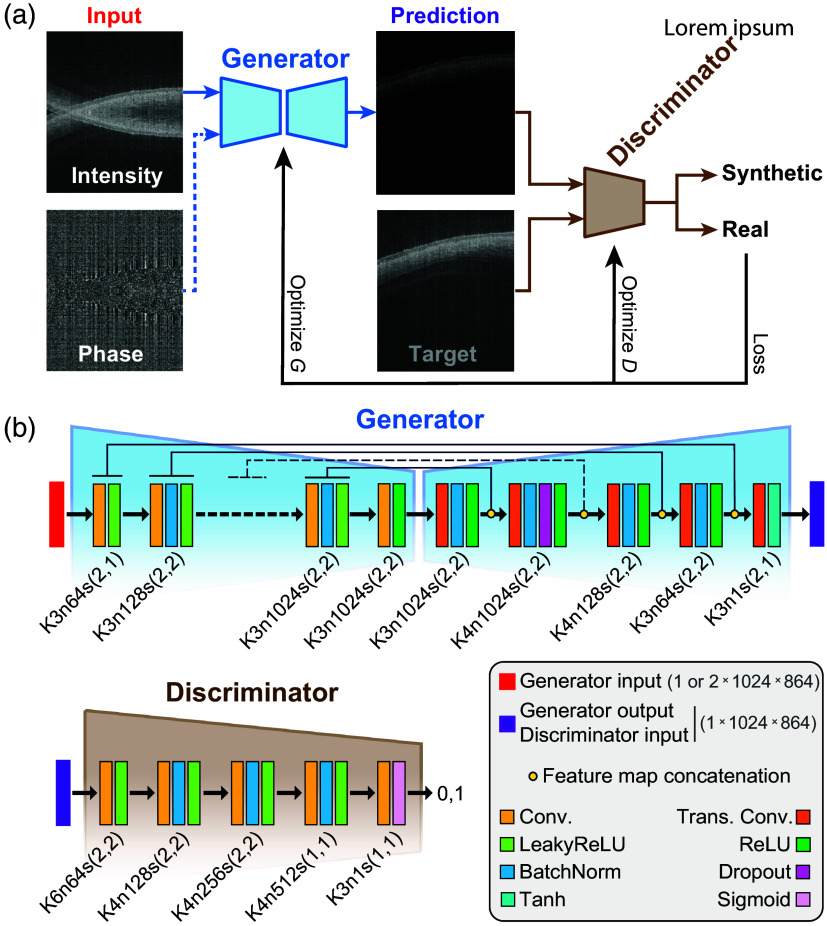
(a) CCR-GAN architecture. (b) Details of generator (G) and discriminator (D) layer’s structure. K, kernel size; n, number of feature maps; S, stride.

The CCR-GAN architecture [Fig. 2(a)] employed in this study was based on the Pix2Pix model,^16^ which was adapted for high-resolution image-to-image translation of size 864×1024  pixels. The generator adopted a U-Net structure with skipped connections and seven hidden layers in the encoder and seven layers in the decoder, whereas the discriminator has five convolutional layers [Fig. 2(b)].

In addition, we modified the input layer of the CCR-GAN model to accept a single-channel input, receiving the intensity image, noted as CCR-GAN-i, as well as a double-channel input, receiving both the intensity and phase images, noted as CCR-GAN-p. The generator (G) and discriminator (D) were trained together, competing in a min–max zero-sum game based on the classification score (0,1) provided by the discriminator’s final layer. Our CCR-GAN objective function $O={\arg\;\min}_{D}\;\max_{G}L_{\mathrm{CCR}-\mathrm{GAN}}(G,D)+\lambda L_{L1}(G)$ consisted of generator and discriminator loss terms, inclusive of a λ weighted $L_{L1}$ loss term. Specifically, the generator loss $L_{G}=\theta_{G}L_{\mathrm{BCE},\mathrm{pred}}$ was represented by a $\theta_{G}$-weighted binary cross-entropy (BCE) loss $L_{\mathrm{BCE},\mathrm{pred}}$ of the generated images. On the other hand, the discriminator loss $L_{D}=\theta_{D}(L_{\mathrm{BCE},\text{real}}+L_{\mathrm{BCE},\mathrm{pred}})/2+L_{\mathrm{PERC}}$ comprised two BCE loss terms of the real and generated images, $L_{\mathrm{BCE},\text{real}}$ and $L_{\mathrm{BCE},\text{real}}$, respectively, weighted with $\theta_{D}$, and a perceptual adversarial loss $L_{\mathrm{PERC}}$ term. The perceptual adversarial loss was defined as the sum of L1 distances between predicted and real images observed at different discriminator layers.^17^ We used Adam optimizer with a learning rate of 0.0001, β values of 0.5 and 0.999, and trained the networks for up to 140 epochs. Generally, GAN training is characterized by instability and losses oscillating throughout the training. Therefore, we selected the best epoch number based on quantitative evaluation metrics on the validation set, even if losses had not strictly converged to a stable point. We trained three distinct models for the three samples.

The image data were split at volumetric scan level for training and testing (Table 1). Specifically, during the training step, we considered a split ratio of 95:5 for training data and validation data.

**Table 1:** Details on the data used to train and test CCR-GAN.

	Train and validation			Test		
	Vol	B-scan	Scanning location	Vol	B-scan	Scanning location
Phantoms	14	2000	Random	5	700	Random
Human skin	14	1900	Palm	5	500	Fingertip
Mouse eye	12	3200	S/N 1 and 2	4	1000	S/N 1 and 3

Vol, volume pair numbers

### 2.3 Statistical Analysis

We utilized peak signal-to-noise ratio (PSNR), structural similarity index (SSIM), and Fréchet inception distance (FID)^18^ to quantitatively assess the performance of CCR-GAN-i and CCR-GAN-p. We represented the mean of the results with box plots together with error bars indicating the corresponding standard deviation. For the FID metric, the error bars were obtained by conducting multiple experimental runs using different subsamples of the data. P values were calculated using F-test with scores less than 0.05 considered statistically significant. All the statistical analysis was performed using Python and the scikit-learn library.

## 3 Results

**Fig. 3:**
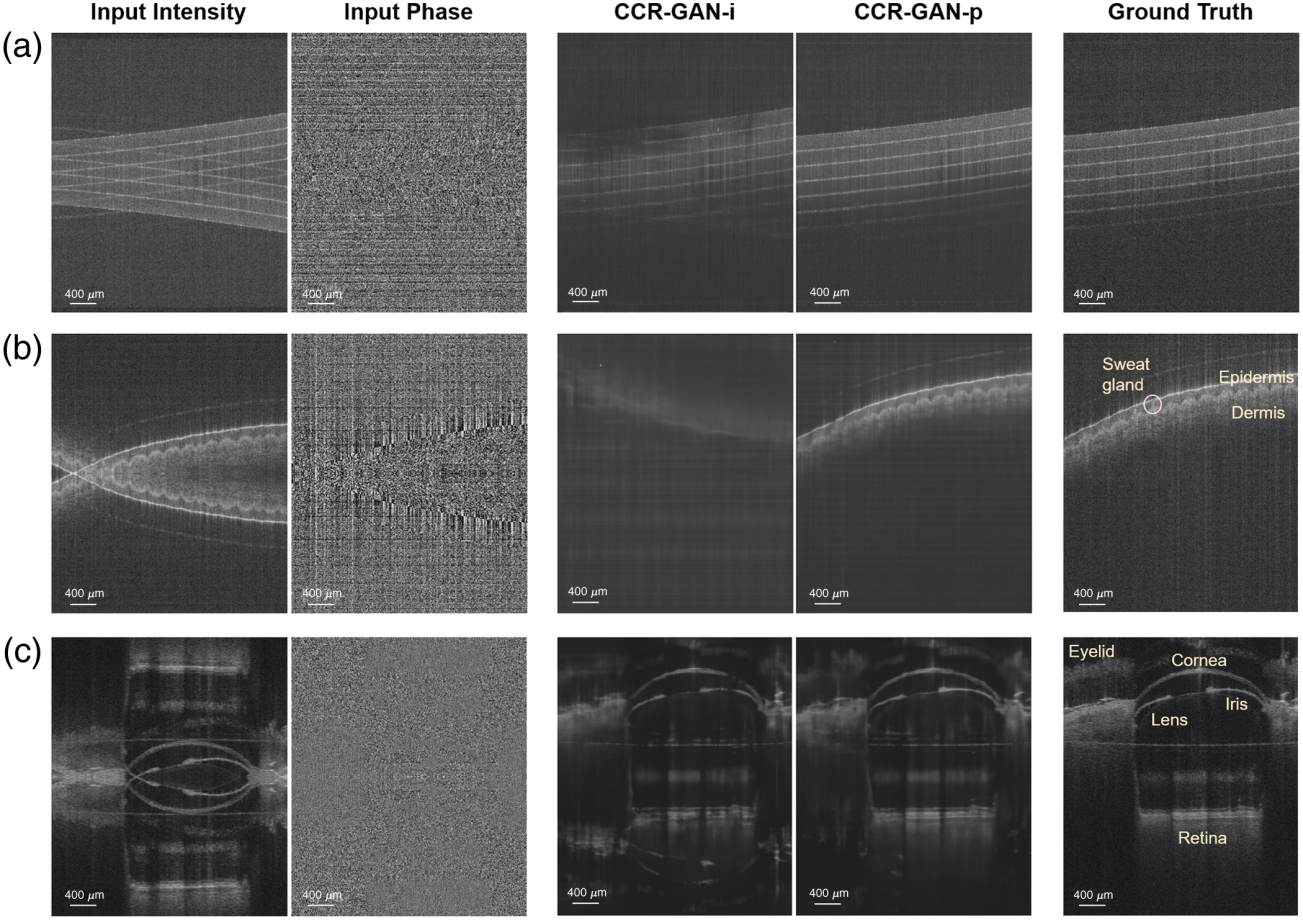
Inputs, CCR-GAN-i and CCR-GAN-p outputs, and ground truth of (a) polymer phantom, (b) human skin, and (c) mouse eye.

**Fig. 4:**
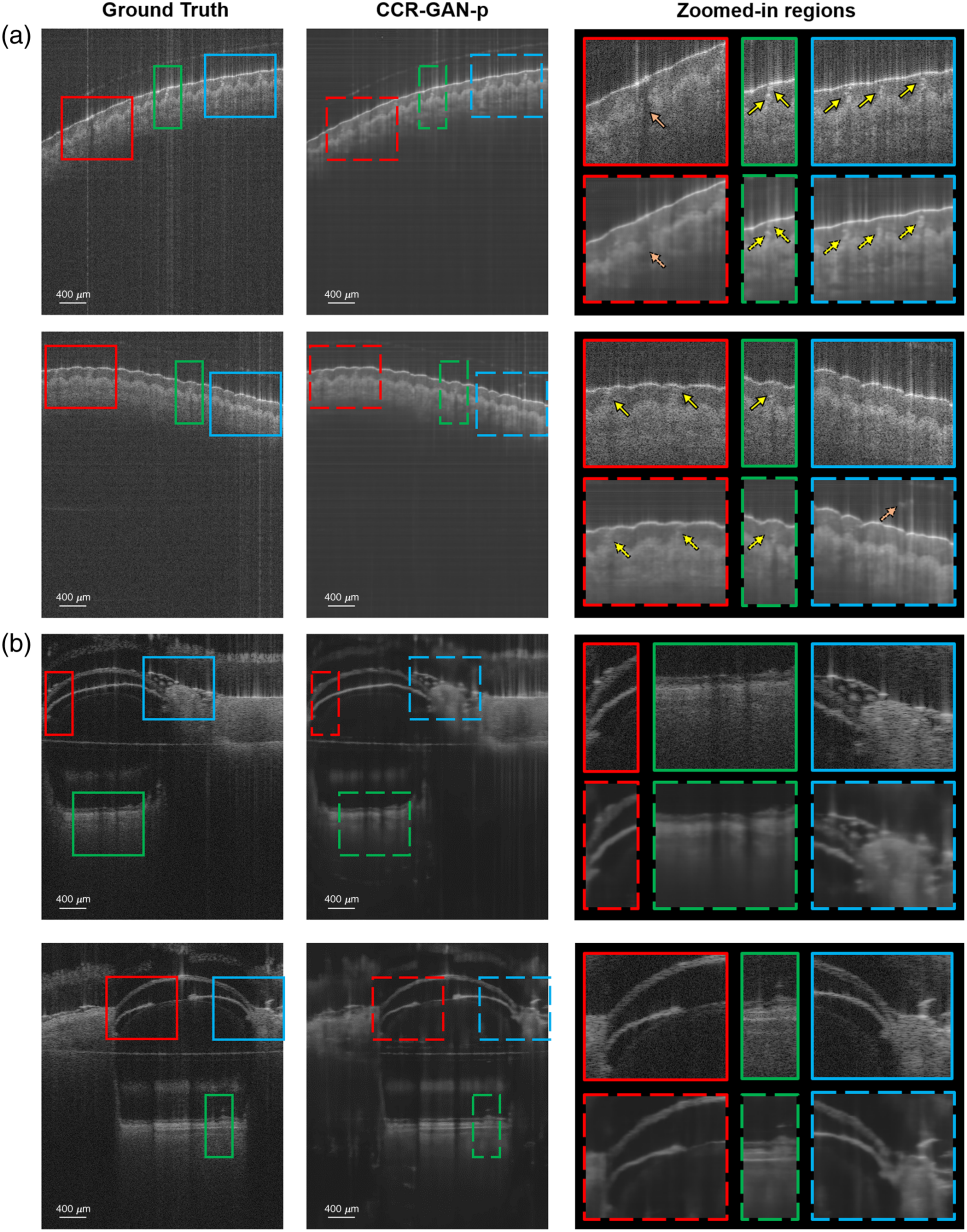
(a) Human skin and (b) mouse eye zoomed-in regions from three areas. Two different samples for each class are represented to compare the CCR-GAN-p results with the respective ground truth images.

The CCR-GAN-i and CCR-GAN-p models, each specifically trained for their respective sample categories, were utilized to process the test dataset and generate images without CCAs (Fig. 3). Upon visual examination, it is evident that CCR-GAN-i could not completely eliminate the CCAs; in contrast, CCR-GAN-p could achieve successful and accurate reconstruction of CCA-free images. Interestingly, we observed that the sample depth position of the generated images was automatically adjusted to be aligned with that of the ground truth. Figure 4 shows examples from human skin and mouse eye samples and includes zoomed-in regions. Overall, we ensured that no significant anatomical features, such as sweat ducts or retinal layers, were altered in shape or position. In addition, we found that the generated images of CCR-GAN-p accurately represented the sample structures of the input images, but the fine speckle patterns were blurred compared with the input and ground truth images upon close inspection. However, in the case of the skin samples, one duct was reconstructed with poor resolution, and an artifact hallucination appeared. These issues are considered minor and do not significantly detract from the overall findings.

**Fig. 5:**
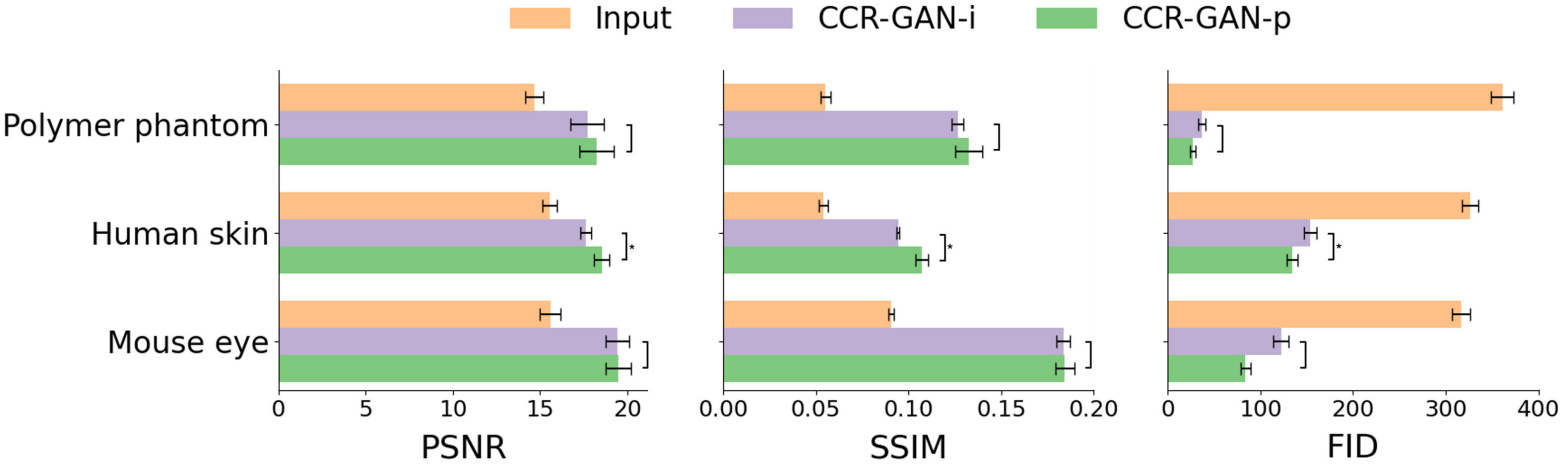
Bar plots of the PSNR, SSIM, and FID scores for the inputs, CCR-GAN-i, and CCR-GAN-p results versus the ground truth. SSIM, structural similarity index metric; FID, Fréchet inception distance. *p<0.5.

We utilized PSNR values, SSIM, and FID scores to quantitatively assess the performance of CCR-GAN-i and CCR-GAN-p (Fig. 5). Lower FID with higher SSIM and PSNR scores mean higher similarity between the generated image and the target image (ground truth). CCR-GAN-p consistently outperformed CCR-GAN-i in terms of PSNR, SSIM, and FID. We found that, for all metrics, only CCR-GAN-i and CCR-GAN-p in the human skin dataset showed a significant difference (p<0.05). Of note, these metrics may not fully reflect the effectiveness of these models because variations in the generated speckle patterns contributed to the metrics but were insignificant for human perception.

## 4 Discussion and Conclusions

In this article, we proposed to use GAN models to remove CCAs for OCT devices to enlarge the imaging range. We demonstrated promising results on various *in vivo* samples including human skin and mouse eyes, confirming the results obtained from simple discrete interfaces in a previous study.^19^ Furthermore, we emphasized the important role of phase images in achieving an effective model, in line with the observations in the previous work reconstructing OCT images from under-sampled^20^ or discontinuous spectral data.^21^

To maintain the integrity of both intensity and phase information, we customized our network to input and output high-resolution data without resizing the original OCT images. We applied standard normalization procedures to stabilize model training. De-normalization of the outputs was applied to reverse the normalization process to obtain the quantitative intensity measurements.

The potential risk of hallucinations when using GANs in medical imaging poses a known challenge for their adoption in clinical practice. Generative deep learning methods have been recently applied to a broad range of tasks, such as frequency-aware super-resolution^22^ and denoising with speckle modulation^23^ in OCT, across various imaging modalities, such as magnetic resonance imaging^24^ and computed tomography,^25^ and other applications.^26^^–^^28^ Recent research demonstrates that such networks can effectively preserve clinically relevant features without introducing significant artifacts, highlighting the potential of these models to become reliable tools in clinical settings.^29^ Figures 3 and 4 showcase some of our generated images. In the skin samples, the epidermis and dermis layers are preserved, and the sweat ducts maintain their original position and shape. In the mouse samples, the retina, cornea, lens, iris, and surrounding tissues are adequately reconstructed. However, one could argue that the sharpness of certain features is decreased, leading to some inaccuracies, such as the duct in Fig. 4. This is primarily due to the blurriness introduced by the GAN model, which affects the fine speckle pattern. In addition, we present an example where a hallucination artifact appeared above the epidermis layer. Overall, these discrepancies do not significantly detract from the key findings, and the CCA was successfully eliminated. The generated images preserve the ground truth of relevant features. Nonetheless, efforts are still required to improve the fidelity of application-specific generative models and to build trust in synthetic images, an area that remains an active focus of research.

Using a GAN architecture instead of a denoising convolutional network allows the model to simultaneously learn to detect the presence of CCA and translate the output to the ground truth by tailoring the loss function. This approach eliminates the need for strict pixel-to-pixel depth position alignment of paired images in the training dataset, rendering it applicable to *in vivo* samples. On the other hand, the variation in gray levels observed in the generated images represents a characteristic of generative models and is a limitation of our approach. Although this variability does not affect the preservation of crucial anatomical features, it may impact the interpretability of absolute intensity values. Future work will focus on refining the model including style loss functions to achieve more consistent intensity.

Variations in generated image speckle patterns, which are often imperceptible to the human eye but still affect metrics such as FID and SSIM, present a challenge when quantitatively assessing the true effectiveness of generative models in OCT data. An effective way to evaluate GAN-generated results remains visual inspection of regions of interest or marking clinically important features, allowing for direct comparison of metrics such as thickness, area, and volume, which are specific to the type of sample.

Our deep learning–based method does not rely on dispersion encoding. Although traditional methods leverage dispersion to locally encode information that aids in resolving the CCA,^30^ our GAN model utilizes holistic spatial and structural information in the OCT image and the inherent symmetry of the CCA to resolve and remove the artifacts. In our OCT, we optimized the optics to minimum unbalanced dispersion between the reference and sample arms and avoided numerical dispersion compensation. In practice, when numerical dispersion compensation is used, the CCA may not manifest as an exact mirror of the sample image but is blurry in the axial direction. The effectiveness of our approaches under such circumstances remains uncertain. In the future, we will explore applying this method on commercially available OCT machines and demonstrate clinical usage such as retinal scans in pathologically elongated eyes. Nevertheless, a further restriction is that the synthetic tomograms may present compromised image sensitivity because the SNR of the ground truth images may be lower than that of the training images due to the SNR rolling off along depth.

**Fig. 6:**
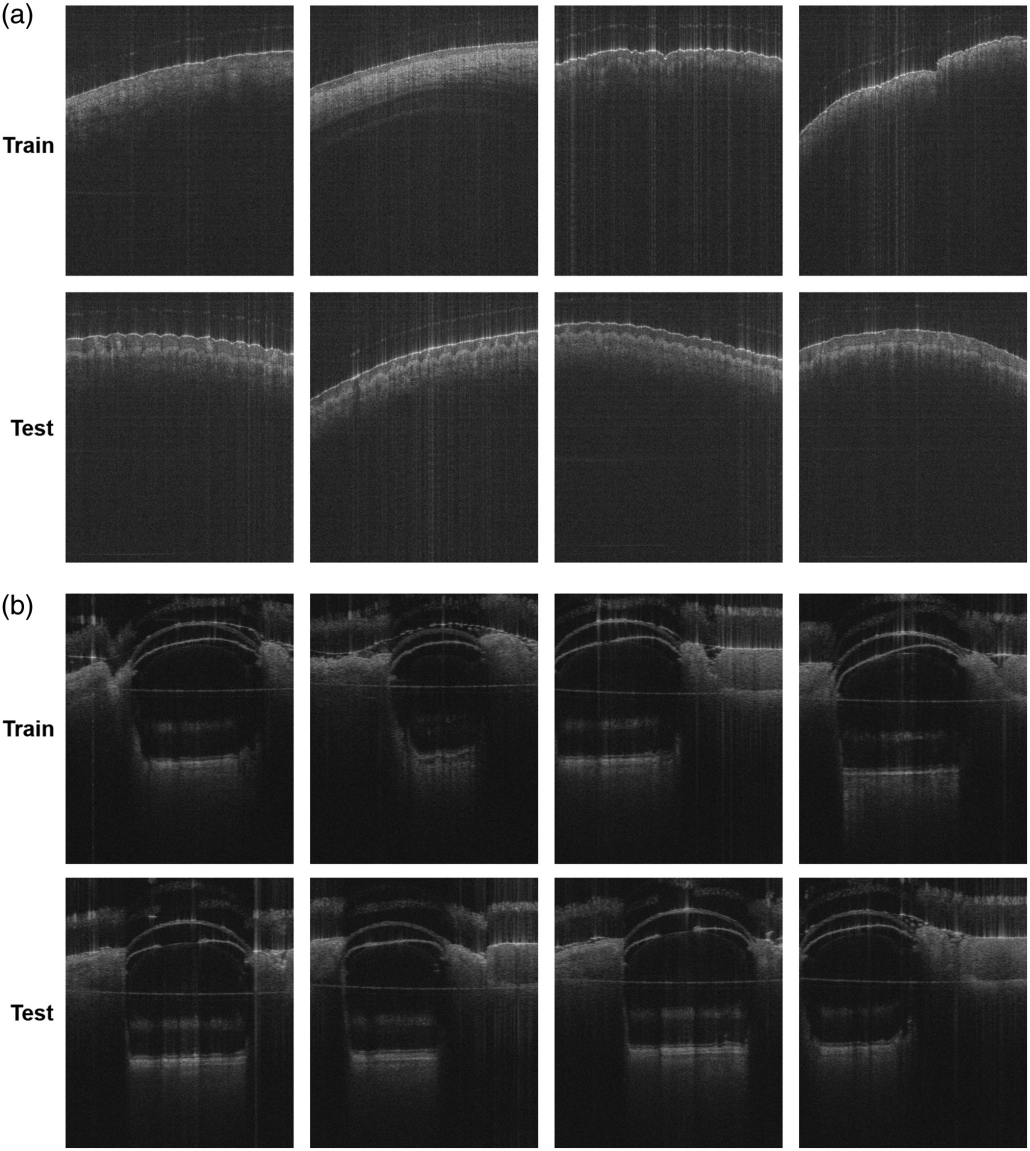
Examples of training and testing images for the *in vivo* samples. (a) Human skin. (b) Mouse eye.

Our generative model needs to be trained with the same species of data, such as skin-to-skin or eye-to-eye, but the network, to perform best, does not involve the exact same sample for training and testing. In Fig. 6, we present a comparison of the training and testing examples for the *in vivo* samples to highlight variations in geometry, features, and scanning areas. We attempted but failed to train a general model as the model could not output any meaningful OCT images. The need to develop separate models for each specific type of sample can be due to the limited dataset size: we speculate that scaling up the size of the model and data would enable a general model to work for a wide range of different sample types. In addition, the unique phase information inherent to each sample, particularly the differences between static and *in vivo* conditions, may also influence the model’s performance, as phase characteristics can vary significantly, impacting generalizability. Future work will focus on experimentally validating these factors.

A limitation of our current approach is the reliance on a single measurement range for both input and reference images, which prevents true extension of the imaging range. Although we can effectively remove the complex conjugate ambiguity, this does not properly address the need for enlarging or doubling the image range to a common full-range imaging.^31^^–^^34^ Future efforts will focus on expanding the model’s capabilities to achieve an increased imaging range, thereby enhancing its applicability to a wider range of OCT imaging scenarios.

The phase component in OCT is not typically used for visual interpretation as it lacks significant visually interpretable information. However, in our study, we found that incorporating phase data during the training stage enhanced the learning process of the model. Although we did not evaluate the reconstruction of phase information in this work, future investigations will focus on analyzing the potential of reconstructing phase data. This could be particularly valuable for applications such as OCT angiography, OCT elastography, and Doppler OCT, where phase information is critical for data processing.

From a methodological view, the success of CCA removal for OCT devices using phase-aware GAN models underscores the potential of DL to bring novel solutions to traditional problems in this field.

## Data Availability

Code and data underlying the results presented in this paper are not publicly available at this time but may be obtained from the authors upon reasonable request.
